# Evaluation of the diagnostic performance of the simple method of computed tomography in the assessment of patients with shoulder instability: a prospective cohort study

**DOI:** 10.1186/s12880-018-0290-4

**Published:** 2018-11-23

**Authors:** Tingting Liu, Jianpeng Ma, Hetao Cao, Dongmei Hou, Lin Xu

**Affiliations:** 1 Department of Medical Imaging, Affiliated Hospital of Nantong University, Shi, Jiangsu Sheng, Nantong, 226001 China; 2 Department of Magnetic Resonance Imaging, Dingbian County People’s Hospital, Dingbian, Yulin, 718600 Shaanxi China; 3 Department of Radiology, PLA general hospital, No.28 Fuxing Road, Haidian District, Beijing, 100000 China

**Keywords:** Bankart lesions, Clinical examination tests, Computed tomography, Traditional radiological images, Twin trial

## Abstract

**Background:**

Physical examinations may reveal the instability of a *glenohumeral* joint but cannot diagnose the bony Bankart lesions. Soft tissue Bankart lesion cannot be visualized on traditional radiogram. Magnetic resonance images have high cost and availability issues. The purpose of the study was to access the diagnostic performance of the Computed Tomography (CT) in the assessment of patients with shoulder instability and to diagnose the Bankart and bony Bankart lesions.

**Methods:**

A total of 145 patients with shoulder instability were included in the study. Patients were subjected to clinical examination tests, traditional radiography, and CT. Two orthopedic surgeons, two engineers (trained in musculoskeletal imaging), and two physiotherapists have analyzed the radiological images, CT scans, and the clinical examination tests respectively. The *Chi-square* test or one-way ANOVA/ Dunnett Multiple comparisons test was performed at 99% of confidence level.

**Results:**

Sensitivity (0.972 ± 0.18 vs. 1, *p* = 0.11) and accuracy (0.942 ± 0.17 vs. 1, *p* < 0.0001, *q* = 3.88) for the clinical examination tests combining the traditional radiological images were same to CT. However, the clinical examination tests combining the traditional radiological images had more inconclusive results (5 vs. 1), false-positive results (6 vs. 5), and false negative results (4 vs. 1) than CT. The area that detects the Bankart and bony Bankart lesions at least one time for CT was higher than that of the clinical examination tests combining the traditional radiological images.

**Conclusion:**

CT should be considered for evaluation in patients with shoulder instability and suspected Bankart and bony Bankart lesions.

**Trial registration:**

Researchregistry3990 dated 15 December 2014 (www.researchregistry.com).

## Background

A *glenohumeral* joint is most frequently dislocated [1]. The recurrent shoulder instability affected patients can develop significant functional deficiencies and symptoms of chronic instability [2]. The reason behind recurrent instability is a glenoid bone loss or the Bankart lesion. Anterior shoulder dislocation due to structural damage to the anterior-inferior labrum with tearing of the anterior capsule is called the Bankart lesion. When it includes osseous fragments, it is called the bony Bankart lesion. The treatment involved in the reattachment of the lesion is arthroscopy with surgical suture [3]. The reason behind the Bankart lesion is a loss of 25% and/or more of the width of the inferior glenoid, which leads to a loss of bony support [4]. The glenoid bone loss is frequent in shoulders with the Bankart lesion [5].

The complexity of the combined motions of the degree of the scapulothoracic and the *glenohumeral* joints creates difficulties in preoperative quantification and identification of the Bankart and bony Bankart lesions for decision making of arthroscopy or open procedures [6]. Surgeons may fail in the surgical repair of the Bankart and bony Bankart lesions if not follow protocol for workup and have addressed a bony problem without being aware of it by the differential diagnosis. Moreover, the management of the Bankart and bony Bankart lesions after a failed surgery could be challenging. Therefore, an adaptation of proper diagnostic modality is crucial in the detection of the Bankart and bony Bankart lesions [7]. The optimal treatment for the Bankart and bony Bankart lesions remains controversial [8]. The accepted diagnostic method of quantifying the Bankart and bony Bankart lesions is not available [9] because physical examinations may reveal instability of the *glenohumeral* joints but cannot diagnose the Bankart or bony Bankart lesions. Generally, orthopedic surgeons have preferred radiography, the Computed Tomography (CT), and magnetic resonance imaging (MRI) in the decision making of surgeries [6].

Soft tissue Bankart lesion cannot be visualized on traditional radiogram. With a Grashey view, an external and internal rotation view, an axillary lateral view, a scapular-Y view, a West Point view, a Stryker notch view, and an apical oblique (Garth) view, the sensitivity of traditional radiography for detection of significant glenoid bone loss is not more than 50% [2]. Moreover, an acute fracture of the glenoid rim is often inaccurately quantified by traditional radiography [10]. Therefore, traditional radiography is underestimated bone defects. MRI has high cost and availability issues [3]. MR images are suggested only if the traditional radiography will fail to provide the necessary information for decision making of surgeries [11]. Therefore, there is a strong need for a consensus on the universally accepted diagnostic method of quantifying the Bankart and bony Bankart lesions.

The goal of the study was to access the diagnostic performance of the simple method of CT in the assessment of patients with shoulder instability and to diagnose the Bankart and bony Bankart lesions. The secondary endpoint of the study was to compare sensitivity and accuracy of the clinical examination tests combining the traditional radiological images with CT for decision making of arthroscopy or open procedures for repair of the bony problem of shoulder(s) at level 2 of evidence (Table 1) without conflict of interest.

**Table 1:** Level of Evidence

Level	Diagnostic study
1	Testing previously developed diagnostic criteria
2	Testing diagnostic criteria with gold standard
3	Review article/Meta-analysis on STARD studies
4	Case study
5	Expert opinion

*STARD* Standards for reporting of diagnostic accuracy studies

## Methods

The study had adhered 2013 Declarations of Helsinki, standards for reporting of diagnostic accuracy studies (STARD) guidelines, and the law of China. The work has been reported in line with the strengthening the reporting of cohort studies in surgery (STROCSS) criteria [12].

### Inclusion criteria

Patients age 18 years and above, with a complaint of progressively reduced shoulder function, pain located at the front and lateral side of the shoulder, load-dependent pain, or/and delocalization of the shoulder available with outpatient setting of the PLA general hospital, China, Affiliated Hospital of Nantong University, China, and Dingbian County People’s Hospital, China from 18 December 2014 to 1 February 2018 were included in the study. Patients who had a bony problem of shoulder(s) and signed an informed consent form were subjected to a pre-index test (the pre-index tests were performed for eligible participants screening purposes).

### Pre-index tests

Functional outcomes were measured in pre-index tests as follows to confirm glenoid bone loss:

### Oxford instability shoulder score

It contains 12 items questionnaires with four responses (0–3). The total score (the sum of 12 items) ranged from 0 (excellent) to 48 (the worst) [13].

### Western Ontario shoulder instability index

It has 21 items questionnaires on a visual analog scale score. The total score (the sum of 21 items) ranged from 0 (the excellent) to 100 (the worst) [13].

### Simple shoulder test score

It consists of 12 questions with dichotomous response options, which are scored 0 or 1. The total score (the sum of 12 items) ranged from 0 (the worst) to 12 (the excellent) [13].

### Disability of the arm, shoulder, and hand score

It is a measurement of physical function and symptoms in patients with musculoskeletal disorders from any condition in any joint in the upper extremity. It has 30-questions (grading 0–3). The average of item scores, subtracting one, and multiplying the result by 25 is the resulting score. It has the range from 0 (no disability) to 100 (extreme disability) [14].

### Exclusion criteria

Patients age younger than 18 years and not signed informed consent form were excluded from the study. Patients who had frozen shoulder and arthritis were excluded from the study. Patients who had oxford instability shoulder score, four and less than four were excluded from the study.

**Fig. 1:**
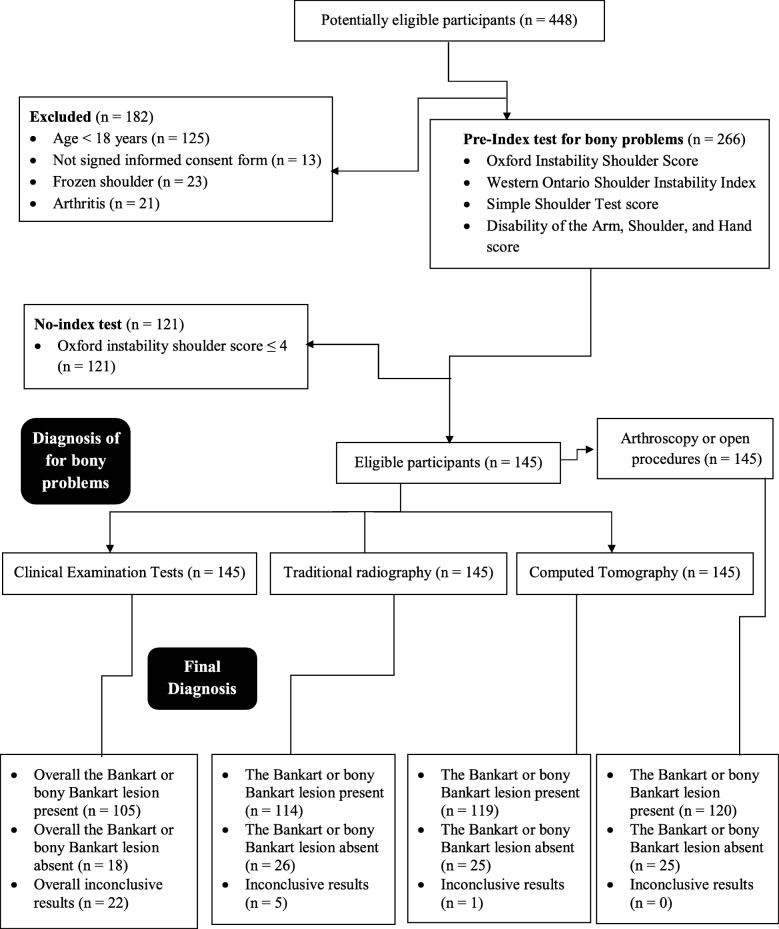
STARD flow diagram of the study

A total of 145 patients with the proved shoulder instability were enrolled in a prospective cohort study. The demographic parameters of the enrolled patients are presented in Table 2. STARD flow diagram of the study is presented in Fig. 1.

**Table 2:** The demographic parameters of the enrolled patients

Characters		Populations
Patients enrolled in the study (sample size)		145
Gender	Male	66 (46)
	Female	79 (54)
Age (years)		28.52 ± 7.56
Reduced shoulder function	DS	114 (79)
	NDS	29 (20)
	Both	2 (1)
Pain located at the front side of the shoulder	DS	117 (81)
	NDS	24 (17)
	Both	4 (2)
Pain located at the lateral side of the shoulder	DS	113 (78)
	NDS	30 (21)
	Both	2 (1)
Load-dependent pain	DS	125 (86)
	NDS	22 (18)
	Both	8 (6)
Delocalization of the shoulder	DS	112 (77)
	NDS	33 (23)
	Both	0 (0)
Incidence of event	The first time	133 (92)
	The second or third time	12 (8)
Functional outcome measures	Oxford Instability Shoulder Score (48–0) ^a^	20.15 ± 1.89
	Western Ontario Shoulder Instability Index (0–100) ^a^	44.57 ± 5.28
	Simple Shoulder Test score (0–12) ^a^	8.52 ± 1.13
	Disability of the Arm, Shoulder, and Hand score (100–0) ^a^	25.47 ± 2.58

Constant data are considered as number (percentage) and continuous data are presented as mean ± SD

^a^The range is reflected as most impaired to the least impaired condition

DS: The dominant side

NDS: The non-dominant side

Oxford Instability Shoulder Score: 0: excellent, 48: The worst

Western Ontario Shoulder Instability Index: 0: excellent, 100: The worst

Simple Shoulder Test score: 0: excellent, 12: The worst

Disability of the Arm, Shoulder, and Hand score: 100: No disability, 0: extreme disability

Enrolled patients had more than one type of demographic characters regarding the bony problem in the shoulder(s)

All patients have China PR origin

### Diagnosis of the Bankart and bony Bankart lesions

#### Clinical examination tests

Physical examinations of shoulders were performed as follows:

#### Apprehension test

The enrolled patients had kept in supine position with the arm in 90^0^ abductions, the elbow in 90^0^ flexions, and maximum external rotation. The evaluator had applied an anterior, external, rotatory force [15].

#### The relocation test

In apprehension test position, the force was applied to the humeral head in the posterior direction [16].

#### The anterior release/ the surprise test

The patient’s affected arm was maintained in the position of apprehension and the pressure on the humeral head was suddenly released by the evaluator [17].

#### The anterior drawer test

Patients were kept in supine positions and affected hand was on the examiner’s axilla, the arm was held in 80–120^0^ of abduction, 0–20^0^ of forwarding flexion, and 0–30^0^ of external rotation. With one hand, the evaluator had stabilized the scapula by applying force on the coracoid process. The other hand was grasped the humeral and drew it out anteriorly [18].

#### The load and shift test/ the push-pull test

Patients were kept in set positions. With the dominant hand, the evaluator was grasped the patient’s elbow and the non-dominant hand (of the evaluator) was grasped the patient’s upper arm. The evaluator was positioned the patient’s arm in 90^0^ of abduction in the scapular plane with no rotation and centered the humeral head of subject on the glenoid by applying a force along the axis of the humeral with the dominant hand to shift the humeral head in the anterior direction [19].

#### The Hyperabduction test

Patients were kept in standing positions, the elbow was flexed at 90^0^, the forearm was horizontal, and the evaluator was standing behind the patients. With the evaluator’s forearm of the dominant hand, the shoulder girdle was pushed down firmly, while the evaluator’s non-dominant hand was lifted the patient’s upper limb, which was relaxed in the abduction [13].

All tests were performed by two physiotherapists (evaluators; three years of experience) who were blinded regarding radiological images and in order of those have written. The interpretation of observations of all the tests for considering them positive or negative are presented in Table 3.

**Table 3:** Interpretation of clinical examination tests

Clinical Examination Tests	Observations regarding patients feeling after the test	
	Considered positive	Considered negative
Apprehension Test	An apprehensive feeling	Only pain noticed
The Relocation Test	Apprehensive feeling or pain is reduced	Apprehensive feeling or pain is not reduced
The Anterior Release/ the Surprise Test	An apprehensive experience	Only pain noticed
The Anterior Drawer Test	Humeral head increased translation than the other shoulder	Only pain noticed
The Load and Shift Test/ the Push-Pull Test	displayed apprehension	No apprehension
The hyperabduction Test	Arm hyper-abducted ≥105^0^	Arm hyper-abducted < 105^0^

Each patient underwent physical examinations total four times by two physiotherapists (blinded regarding radiological images)

### Traditional radiographs

**Fig. 2:**
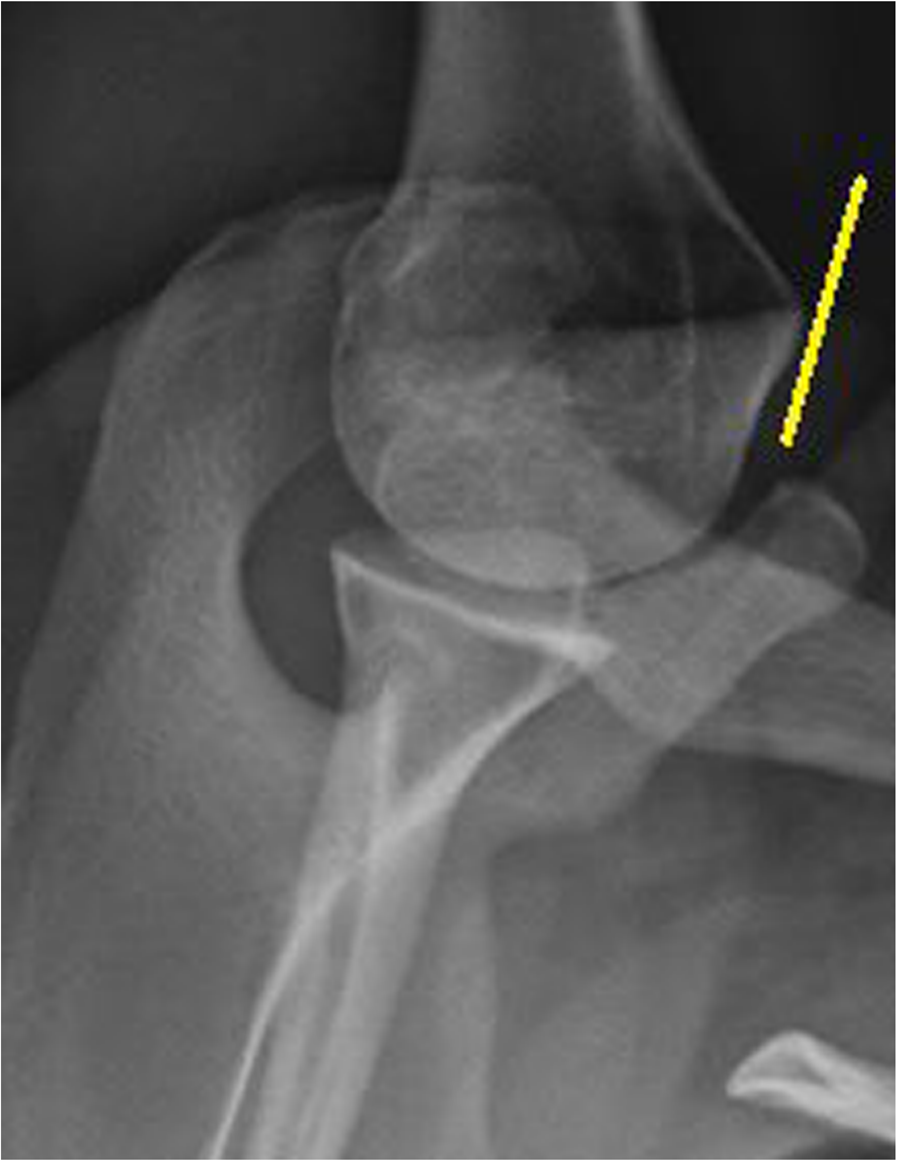
Traditional radiological image of the shoulder with the Bankart lesions. The yellow line indicates the Bankart lesions. The Grashey view of the patient’s shoulder. Images were checked by two radiologists

All enrolled patients were subjected to axillary traditional radiographs (GE Healthcare, Chicago, IL, USA) at an axial position and at 90^0^. Conventional axillary lateral, anteroposterior (in exo-rotation or in endo-rotation) radiographs and the Grashey view of the affected shoulder were performed (Fig. 2).

### CT scans

**Fig. 3:**
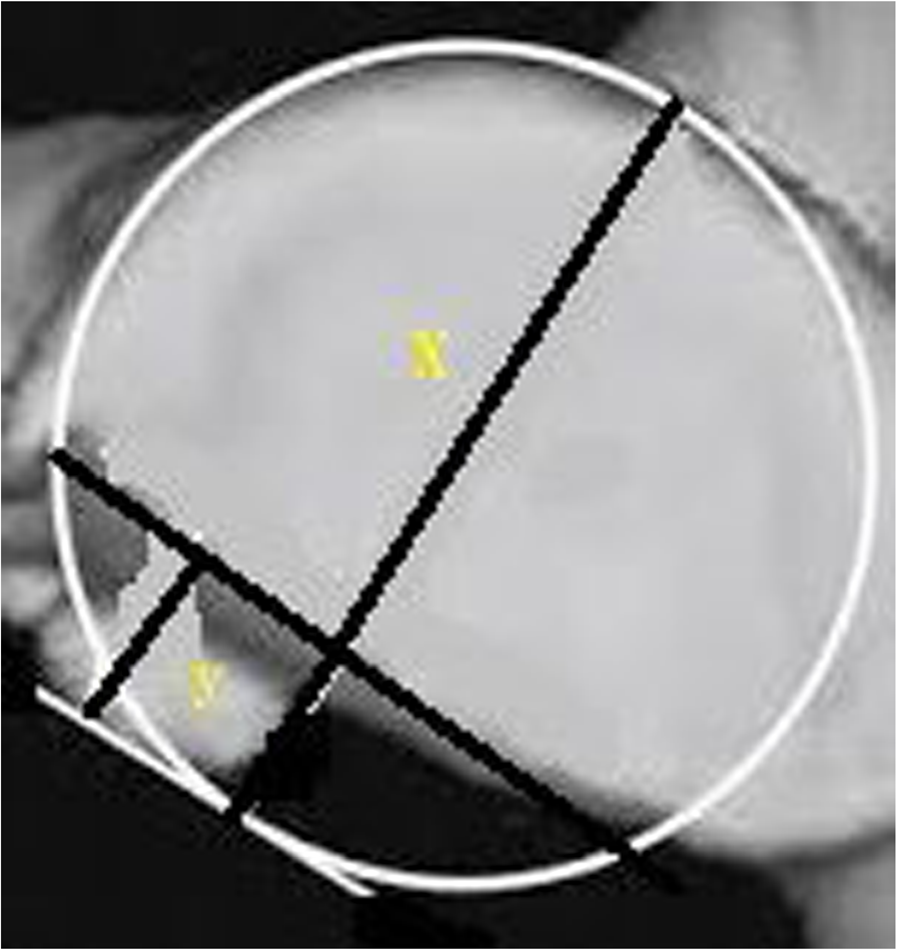
The Computed Tomography of the patient’s shoulder with the Bankart lesions. X: The inferior glenoid circle. Y: The eroded missing area. Images were checked by two engineers (diploma course in musculoskeletal imaging assessments in PR. China after MD radiology) trained in musculoskeletal imaging

Spiral CT (CATIA, France) was performed at 1.25 mm thickness and 0.625 mm interval of affected shoulder to confirm or exclude the Bankart and Bony Bankart lesions (Fig. 3).

### Image analysis

**Fig. 4:**
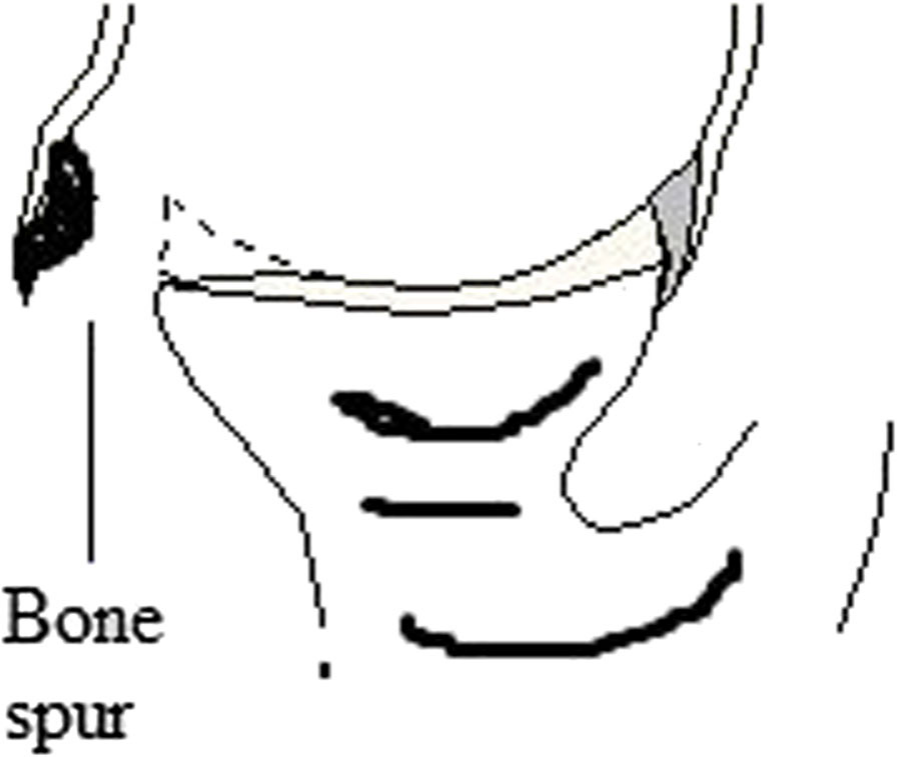
Traditional radiological images method for detection of the Bankart or bony Bankart lesions. Images were checked by two radiologists. A small bone spur in traditional radiological images of the shoulder was considered as the Bankart or bony Bankart lesions present

**Fig. 5:**
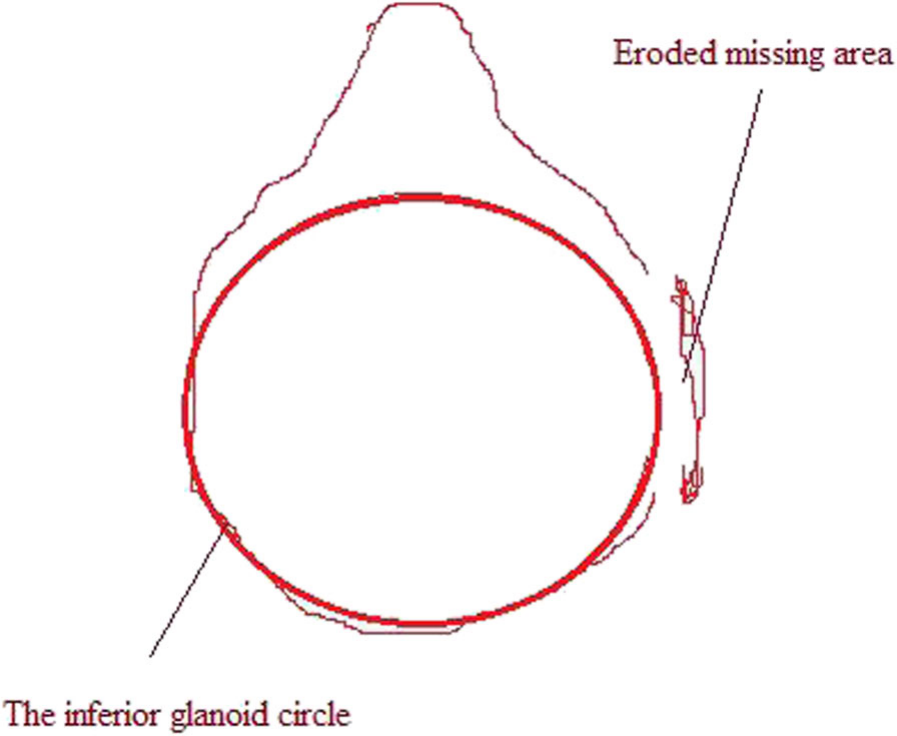
Surface area method of the simple Computed Tomography in the assessment of patients with shoulder instability and to diagnose the Bankart and bony Bankart lesions. Images were checked by two engineers (diploma course in musculoskeletal imaging assessments in PR. China after MD radiology) trained in musculoskeletal imaging. The Bankart and/or bony Bankart lesions was confirmed if the percentage missing area was minimum 20%

Radiological images were checked by a radiologist (three years of experience) under the lightbox (Efftronics Systems Pvt. Ltd., China). A small bone spur in traditional radiological images of the shoulder was considered as the Bankart or bony Bankart lesions present (Fig. 4). The surgeons had decided the Bankart or bony Bankart lesions and taken the decision of arthroscopy or open procedures. CT scans were evaluated using CATIA (CATIA V5R21; Dassault Systemes, France). The percentage missing area was calculated by surface area missing and the inferior glenoid circle (Fig. 5) as per Eq. 1 (surface area method) [20]. The Bankart and/or bony Bankart lesions was confirmed if the percentage missing area was minimum 20% [3].

1 $$ \mathrm{The}\ \mathrm{percentage}\ \mathrm{missing}\ \mathrm{area}=\frac{\mathrm{The}\ \mathrm{eroded}\ \mathrm{missing}\ \mathrm{area}}{\mathrm{The}\ \mathrm{inferior}\ \mathrm{glenoid}\ \mathrm{circle}}\times 100 $$

Surgery curative was considered as ‘gold standard’ in all the operative case. If any kind of bony problem (e.g. inflammation, rupture, ligament straitening, Hill–Sachs lesion) had found in diagnosis, which was lead to arthroscopy or opens surgical procedure and actually it was the Bankart and/or bony Bankart lesions considered as a ‘false negative’. If the Bankart and/or bony Bankart lesions had found in diagnosis, which was lead to surgery and actually it was the other than the bony problem (frozen shoulder, arthritis, Hill–Sachs lesion) considered as a ‘false positive’. The accurate Bankart and bony Bankart lesions absent category were also found in all diagnosis because all patients who had to complain of the shoulder(s) instability were included and there is no any accurate clinical test until the time to confirm the Bankart and bony Bankart lesions.

### Beneficial analysis of diagnostic methods

Decision-making curve for detection of the Bankart and bony Bankart lesions was drawn by decision curve analysis as per Eqs. 2 and 3 [21]:

2 $$ \mathrm{Decision}\ \mathrm{making}\ \mathrm{of}\ \mathrm{the}\ \mathrm{Bankart}\ \mathrm{and}\ \mathrm{bony}\ \mathrm{Bankart}\ \mathrm{lesions}=\mathrm{True}\ \mathrm{Positive}\ \mathrm{ratio}\ \mathrm{of}\ \mathrm{the}\ \mathrm{Bankart}\ \mathrm{and}\ \mathrm{bony}\ \mathrm{Bankart}\ \mathrm{lesions}-\left(\mathrm{False}\ \mathrm{positive}\ \mathrm{ratio}\ \mathrm{of}\ \mathrm{the}\ \mathrm{Bankart}\ \mathrm{and}\ \mathrm{bony}\ \mathrm{Bankart}\ \mathrm{lesions}\times \mathrm{Weighting}\ \mathrm{factor}\right) $$

3 $$ \mathrm{Weighting}\ \mathrm{factor}=\frac{\mathrm{A}\ \mathrm{level}\ \mathrm{of}\ \mathrm{diagnostic}\ \mathrm{confidence}\ \mathrm{above}\ \mathrm{it}\ \mathrm{arthroscopy}\ \mathrm{or}\ \mathrm{opens}\ \mathrm{surgical}\ \mathrm{procedure}\ \mathrm{had}\ \mathrm{performed}\ }{1-\mathrm{A}\ \mathrm{level}\ \mathrm{of}\ \mathrm{diagnostic}\ \mathrm{confidence}\ \mathrm{above}\ \mathrm{it}\ \mathrm{arthroscopy}\ \mathrm{or}\ \mathrm{opens}\ \mathrm{surgical}\ \mathrm{procedure}\ \mathrm{had}\ \mathrm{performed}} $$

Weighting factor: The risk of overdiagnosis.

### Inter-and intra-observer reliability

For purposes of the reliability of assessments, two orthopedic surgeons were analyzed twice times the traditional radiological images, two engineers trained in musculoskeletal imaging assessments (this is diploma course for musculoskeletal imaging assessments in PR. China after MD radiology; three years of experience in image analysis) were evaluated the CT scans twice, and two physiotherapists were evaluated clinical examination tests twice (each patient underwent physical examinations four times). All were blinded to the others’ conclusions.

### Cost of diagnosis

Cost of diagnosis was included hospitalization, charges of experts, and radiographers.

### Statistical analysis

Kappa statistics (considering kappa value (k) ≤ 0.4 low, 0.41–0.6 moderate, 0.61–0.8 substantial, and ≥ 0.81 perfect inter-and intra-observer reliabilities) were used to determine reliability [22]. One-way analysis of variance (ANOVA) following Dunnett Multiple comparisons tests (considering critical value (*q*) > 4.148 as significant) was performed to compare constant diagnostic parameters [23] and the *Chi-square* test was performed for categorical data. All results were considered significant at 99% of confidence level. InStat (vWindow, GraphPad, IL, USA) was used for statistical analysis.

## Results

Functional outcome measures were strongly correlated with the Bankart and bony Bankart lesions. k for physiotherapists, orthopedic surgeons, and trained engineers (in musculoskeletal imaging) were greater than 0.81 indicated perfect inter-and intra-observer reliabilities (Table 4).

**Table 4:** Reliability of assessments

Parameters	Overall Clinical examination tests	The traditional radiological images	Computed Tomography
Sample size	145	145	145
Evaluators	Physiotherapists	Orthopedic surgeons	^a^Engineers trained in musculoskeletal imaging
Tested Material	Shoulders of subjects	Traditional radiological images	Computed Tomography images
Numbers of evaluators	2	2	2
Numbers of test/images performed	4	1	1
Numbers of test/images analyzed	4	2	2
Tool for decision making	1 × 1 table	Lightbox	CATIA V5R21 software
^b^The method adopted	Apprehension tests	A small bone spur in images	The glenoid ratio method (% missing area ≥ 20%)
kappa value	0.811	0.825	0.891

kappa value ≤0.4 low; 0.41–0.6 moderate; 0.61–0.8 substantial; and ≥ 0.81 perfect inter-and intra-observer reliabilities

^a^Diploma course in musculoskeletal imaging assessments in PR. China after MD radiology

^b^for decision making of the Bankart lesion

**Fig. 6:**
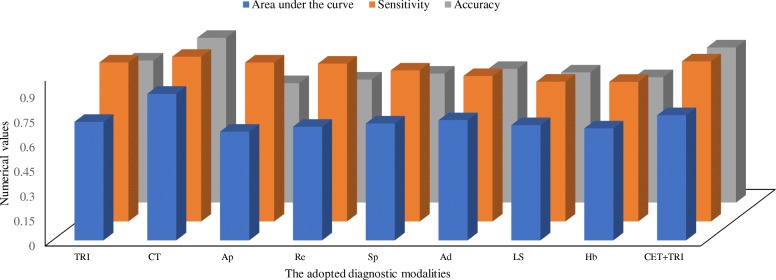
The analogy of diagnostic modalities for decision making of surgeries of the Bankart and bony Bankart lesions. Accuracy and sensitivity of Computed Tomography were considered as 1. One-way repeated-measures ANOVA following Dunnett Multiple comparisons tests was used for statistical analysis. A *p*-value < 0.01 and *q*-value > 4.148 were considered significant. TRI: The traditional radiological images, CT: The Computed Tomography, Ap: Apprehension test, Re: Relocation test, Sp: Surprise test, Ad: Anterior drawer test, LS: Load and shift test, Hb: Hyperabduction test, CET + TRI: Clinical examination tests combining the traditional radiological images

Among clinical examination tests, surprise test has high sensitivity (0.917 ± 0.15) and the anterior drawer test has high accuracy (0.811 ± 0.18). The area under the curve for the clinical examination tests combining the traditional radiological images was fallen down than CT (0.76 ± 0.11 vs. 0.89 ± 0.14, *p* < 0.0001, *q* = 9.39). Overall, the clinical examination tests combining the traditional radiological images had provided limited information for decision making of surgeries (Fig. 6). With respect to CT, sensitivity for the clinical examination tests combining the traditional radiological images had no statistical significance difference (0.972 ± 0.18 vs. 1, *p* = 0.11) even for accuracy there were no statistical significance difference (0.942 ± 0.17 vs. 1, *p* < 0.0001, *q* = 3.88). However, the clinical examination tests combining the traditional radiological images had more inconclusive results (5 vs. 1), false-positive results (6 vs. 5), and false negative results (4 vs. 1) than CT (Table 5). CT was flawless in the decision making of surgeries for the Bankart and bony Bankart lesions than the clinical examination tests combining the traditional radiological images (Table 6).

**Table 5:** Parameters of the adopted diagnostic methods for evaluation of the Bankart and bony Bankart lesions

Parameters	CET						TRI (7)	CET + TRI (9)	CT (10)	SA 9 vs. 10	
	Ap (1)	Re (2)	Sp (3)	Ad (4)	LS (5)	Hb (6)					
Sample size	145	145	145	145	145	145	145	145	145	*p*	*q*
BL present	77 (53)	81 (56)	87 (60)	93 (64)	91 (62)	88 (60)	99 (69)	108 (75)	114 (78)	0.136	N/A
BL absent	23 (16)	22 (15)	21 (15)	19 (13)	18 (12)	17 (12)	20 (14)	22 (15)	24 (17)	0.792	N/A
F (+ve)	36 (25)	31 (21)	19 (13)	9 (6)	6 (4)	8 (6)	15 (10)	6 (4)	5 (3)	0.056	N/A
F (−ve)	3 (2)	4 (3)	5 (3)	7 (5)	8 (6)	9 (6)	6 (4)	4 (3)	1 (1)	0.057	N/A
I/C	6 (4)	7 (5)	13 (9)	17 (12)	22 (16)	23 (16)	5 (3)	5 (3)	1 (1)	0.0286	N/A
AUC	0.66 ± 0.08	0.69 ± 0.05	0.71 ± 0.08	0.73 ± 0.04	0.71 ± 0.07	0.68 ± 0.06	0.72 ± 0.1	0.76 ± 0.11	0.89 ± 0.14	< 0.0001	9.39
Sensitivity	0.91 ± 0.16	0.96 ± 0.06	0.917 ± 0.15	0.88 ± 0.08	0.85 ± 0.08	0.68 ± 0.07	0.97 ± 0.15	0.972 ± 0.18	1	0.11	N/A
Accuracy	0.72 ± 0.15	0.75 ± 0.07	0.78 ± 0.05	0.811 ± 0.18	0.79 ± 0.09	0.76 ± 0.07	0.86 ± 0.14	0.942 ± 0.17	1	< 0.0001	3.88

Constant data are represented as number (percentage)

Surgery curative was considered as ‘gold standard’

*Chi-square* independence test for constant data and One-way repeated-measures ANOVA following Dunnett Multiple comparisons tests for continuous data were used for statistical analysis

A *p*-value < 0.01 was considered significant

A *q*-value > 4.148 was considered significant

*N/A* Not applicable, *BL* Accurate the Bankart and bony Bankart lesions, *I/C* Inconclusive results, *AUC* Area under the curve, *Ap* Apprehension test, *Re* Relocation test, *Sp* Surprise test, *Ad* Anterior drawer test, *LS* Load and shift test, *Hb* Hyperabduction test, *TRI* The traditional radiological images, *CET* Clinical Examination Tests, *CET + TRI* Clinical Examination Tests combining the traditional radiological images, *CT* The Computed Tomography, *SA* Statistical analysis

F (+ve): False positive: The Bankart and/or bony Bankart lesions had found in diagnosis, which was led to surgery and actually it was the other bony problem

F (−ve): False negative: A bony problem had found in diagnosis, which was lead arthroscopy or open surgical procedure and actually it was the Bankart and/or bony Bankart lesions

**Table 6:** Performance parameters results of the adopted diagnostic methods for evaluation of the Bankart and bony Bankart lesions

Diagnostic modalities	True positive the Bankart and bony Bankart lesions ratio	False positive the Bankart and bony Bankart lesions ratio
Computed Tomography	0.79	0.03
Traditional radiology	0.68	0.10
Without diagnosis images	0.6	0.2
Apprehension test	0.53	0.25
Relocation test	0.56	0.21
Surprise test	0.6	0.13
Anterior drawer test	0.64	0.06
Load and shift test	0.63	0.04
Hyperabduction test	0.61	0.06
Clinical Examination Tests combining the traditional radiological images	0.75	0.15

**Fig. 7:**
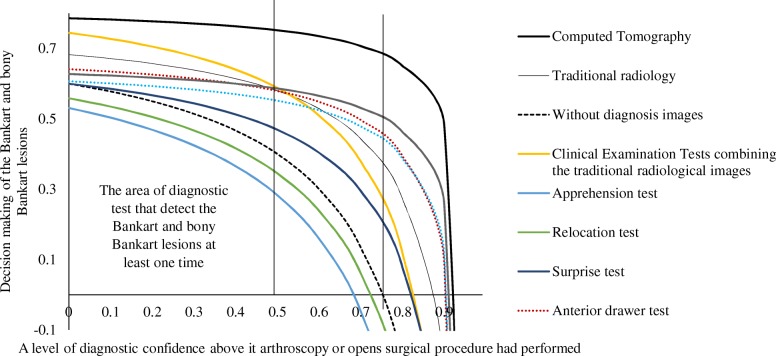
Decision-making curve analysis for detection of the Bankart and bony Bankart lesions. Radiological images were checked by two radiologists. The Computed Tomographic images were checked by two engineers (diploma course in musculoskeletal imaging assessments in PR. China after MD radiology) trained in musculoskeletal imaging. Two physiotherapists evaluated clinical examination tests twice

The area that detects the Bankart and bony Bankart lesions at least one time for CT was higher than that of clinical examination tests combining the traditional radiological images (Table 7). At 0–50% level of diagnostic confidence, orthopedic surgeons had preferred the clinical examination tests combining the traditional radiological images but these tests did not give any sufficient information for decision making of surgery. However, above 50% of the level of diagnostic confidence for the clinical examination tests combining the traditional radiological images, patients had a risk of overdiagnosis and overtreatment. In the range of 0–75% of the level of diagnostic confidence, CT was the most reliable imaging modality and above 75% of the level of diagnostic confidence, CT had a risk of overdiagnosis and overtreatment (Fig. 7).

**Table 7:** Beneficial score analysis of the adopted diagnostic methods for evaluation of the Bankart and bony Bankart lesions

		Decision making of the Bankart and bony Bankart lesions									
CP	WF	CT	TRI	WDC	CET						
					Ap	Re	Sp	Ad	LS	Hb	CET + TRI
0	0	0.79	0.68	0.6	0.53	0.56	0.6	0.64	0.63	0.61	0.75
0.1	0.11	0.78	0.67	0.58	0.50	0.53	0.59	0.63	0.62	0.60	0.73
0.2	0.25	0.78	0.66	0.55	0.47	0.51	0.57	0.63	0.62	0.59	0.71
0.3	0.43	0.77	0.64	0.51	0.43	0.47	0.54	0.61	0.61	0.58	0.68
0.4	0.67	0.76	0.61	0.47	0.37	0.42	0.51	0.60	0.60	0.57	0.64
0.5	1	0.75	0.58	0.4	0.28	0.35	0.47	0.60	0.59	0.55	0.59
0.6	1.5	0.74	0.53	0.3	0.16	0.24	0.40	0.55	0.57	0.52	0.51
0.7	2.33	0.71	0.44	0.13	−0.05	0.06	0.29	0.50	0.53	0.48	0.38
0.8	4	0.65	0.27	−0.2	−0.46	−0.3	0.08	0.39	0.46	0.39	0.12
0.9	9	0.48	−0.24	−1.2	−1.70	−1.37	− 0.58	0.082	0.26	0.11	−0.67
0.99	99	−2.58	−9.51	−19.2	−24.05	−20.61	−12.37	−5.50	−3.47	−4.86	− 14.82

*CP* A level of diagnostic confidence above its arthroscopy or opens surgical procedure had performed, *WF* Weighting factor (risk of overdiagnosis), *CT* The Computed Tomography, *TRI* The traditional radiological images, *Ap* Apprehension test, *Re* Relocation test, *Sp* Surprise test, *Ad* Anterior drawer test, *LS* Load and shift test, *Hb* Hyperabduction test, *CET* Clinical examination tests, *CET + TRI* Clinical examination tests combining the traditional radiological images, *WDC* Without diagnosis images or Clinical Examination Tests

$$ \mathrm{Weighting}\ \mathrm{factor}=\frac{\mathrm{A}\ \mathrm{level}\ \mathrm{of}\ \mathrm{diagnostic}\ \mathrm{confidence}\ \mathrm{above}\ \mathrm{its}\ \mathrm{arthroscopy}\ \mathrm{or}\ \mathrm{opens}\ \mathrm{surgical}\ \mathrm{procedure}\ \mathrm{had}\ \mathrm{performed}}{1-\mathrm{A}\ \mathrm{level}\ \mathrm{of}\ \mathrm{diagnostic}\ \mathrm{confidence}\ \mathrm{above}\ \mathrm{its}\ \mathrm{arthroscopy}\ \mathrm{or}\ \mathrm{opens}\ \mathrm{surgical}\ \mathrm{procedure}\ \mathrm{had}\ \mathrm{performed}} $$

Decision making of the Bankart and bony Bankart lesions = True positive ratio − (weighting factor × false positive ratio)

**Fig. 8:**
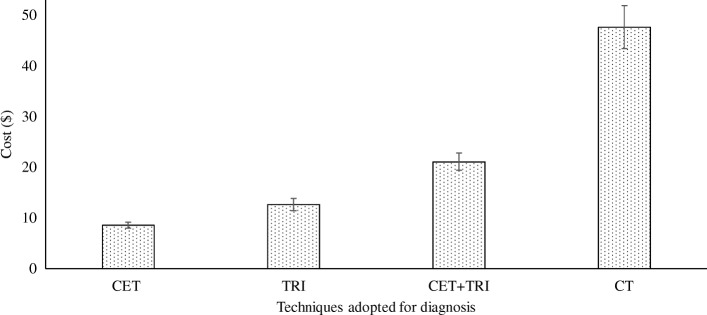
Costs of techniques for the diagnosis of the Bankart and bony Bankart lesions. CET: Clinical examination tests, TRI: The traditional radiological images, CET + TRI: Clinical examination tests combining the traditional radiological images, CT: The Computed Tomography. Data were represented as mean ± SD. Patients enrolled in the study (sample size: n): 145. One-way ANOVA following the Dunnett Multiple comparisons tests was used for statistical analysis. A *p*-value < 0.01 and *q*-value > 4.148 were considered significant

The costs of clinical examination tests, the traditional radiological images, clinical examination tests combining the traditional radiological images, and CT were 8.59 ± 0.59 $/patient, 12.64 ± 1.21 $/patient, 21.11 ± 1.68 $/patient, and 47.65 ± 4.24 $/patient respectively. The cost of CT examinations was higher than the clinical examination tests combining the traditional radiological images (*p* < 0.0001, *q* = 95.05, Fig. 8).

**Fig. 9:**
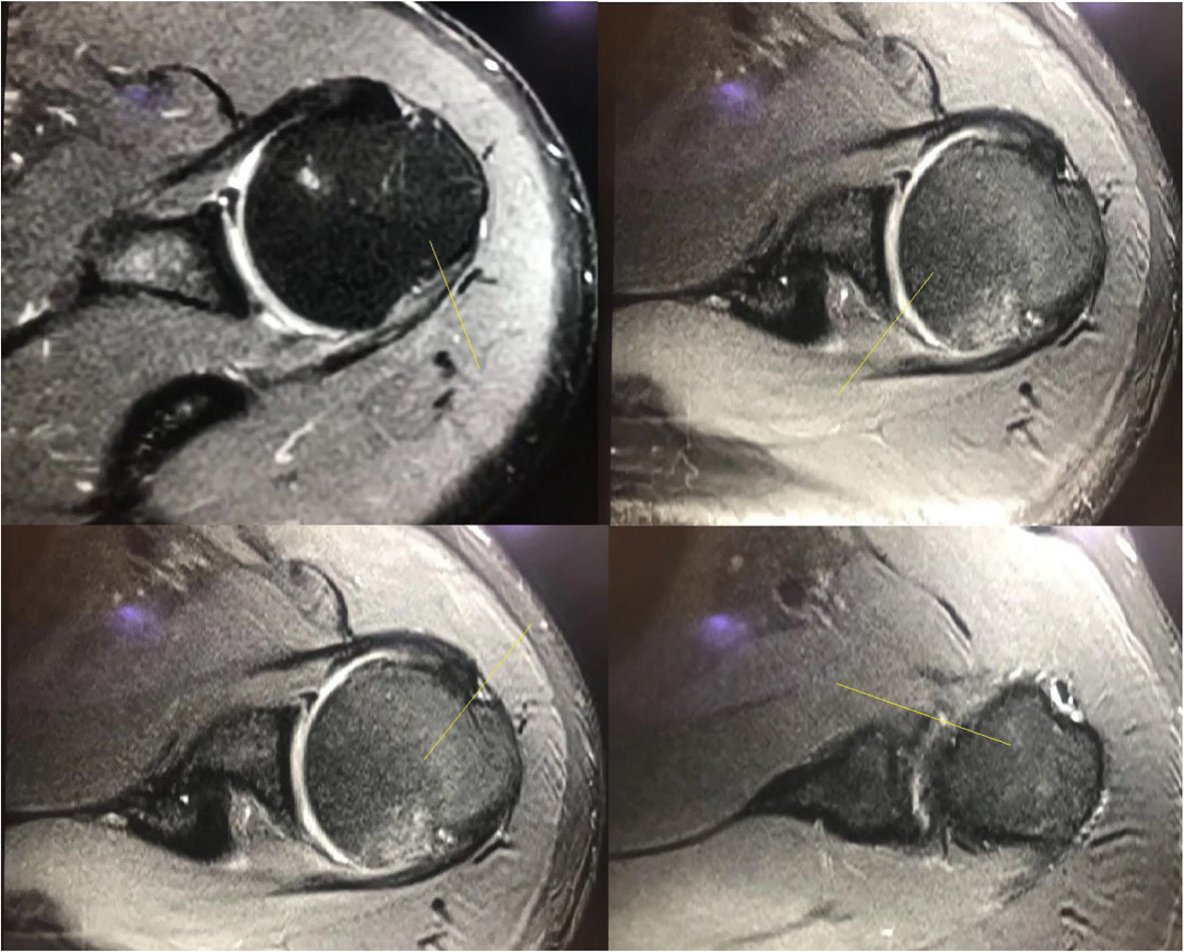
Postsurgical radiographic images of 29-years female patient with shoulder instability showed the union of the grafts during follow-up. Supine view. The yellow line indicates the union of the Bankart lesions

After surgeries radiographs showed the union of the grafts and no incidents of re-dislocation during the follow-up period (Fig. 9).

## Discussion

In the study, clinical examination tests alone were not provided sufficient information and surgeons were preferred radiological images for decision making of surgeries. There are high varieties of diagnostic modalities available for diagnosis of the instability of shoulders. Among the available methods, apprehension tests are the most frequently used combing traditional radiological images [24]. The Bankart and bony Bankart lesions are difficult to diagnose and there are more chances for failure of surgical procedures [25] because of the low sensitivity of radiological modalities [23]. Moreover, the adoption of the diagnostic method before the surgical procedure for the repair of the bony problem(s) of the shoulder is based on a number of dislocations and prior shoulder trauma [13]. The results of the study were not in line with an available study [13] but in line with the review article [26]. Clinical examination tests combing traditional radiological images have limited information to guide the surgeons in the decision making of surgeries for repair of the bony problem(s) of the shoulder [15]. In respect to the decisions of surgeons during the study, clinical examination tests combing traditional radiological images are failed in finding limitations of CT for quantification of the Bankart and bony Bankart lesions.

In the study, CT was provided the best visualization and clinical examination tests combing traditional radiological images were provided insufficient details of the Bankart and bony Bankart lesions for decision making of surgeries. This was because clinical examination tests combing traditional radiological images are failed to provide exact loss of the inferior glenoid [27]. These results were in line with available diagnostic studies [3, 28]. A retrospective study [29], literature reviews [4, 5, 25, 27], and a simulated study [30] are also concluded the same. But the published study is on fewer subjects (sample size: 70) [3] and more numbers of observers (four observers) [3, 28], which have less intra-observer and inter-observer reliabilities [22]. However, the results were not in line with the quantitative assessment of radiography and CT [30]. In respect to the results of the twin study, CT assessments have good agreements for decision making of surgeries of the Bankart and bony Bankart lesions.

CT method was the increased financial burden of the patients. The traditional radiological images and clinical examination tests are cheaper than CT for diagnosis of the Bankart and bony Bankart lesions. However, CT has also prevented the future recurrence and/or failure of surgeries by exact diagnosis [3]. Further research is required to justify the selection of CT for the diagnosis of the Bankart and bony Bankart lesions.

In the limitations of the study, for examples, the study was limited to diagnose the Bankart and bony Bankart lesions only no other type of shoulder delocalization was addressed in the study. The study was not differentiated the Bankart and bony Bankart lesions. The evaluators of clinical examination tests were not blind to the demographic parameters of the enrolled patients. Imaging modalities like CT arthrography, MRI, MRI arthrography provide better soft tissue details compare to CT alone. The lack of ‘gold standard’ or further MRI. The possible justification is that further MRI only for head-to-head comparisons of diagnostic methods is vague in clinical practice. Therefore, researchers have not performed Further MRI.

## Conclusion

The study concluded that the Computed Tomography has better performance parameters than clinical examination tests combining the traditional radiological images and should be considered for evaluation in patients with shoulder instability and the suspected Bankart and bony Bankart lesions.

## Abbreviations

ANOVA: Analysis of variance
CT: Computed Tomography
k: kappa value
MRI: Magnetic resonance imaging
STARD: Standards for reporting of diagnostic accuracy studies
STROCSS: The strengthening the reporting of cohort studies in surgery
